# Nano Methotrexate versus Methotrexate in Targeting Rheumatoid Arthritis

**DOI:** 10.3390/ph16010060

**Published:** 2022-12-31

**Authors:** Heba F. Salem, Marwa Mohamed Abd El-Maboud, Amira S. A. Said, Mohamed Nabil Salem, Dina Sabry, Nadia Hussain, Omnia A. M. Abd El-Ghafar, Raghda R. S. Hussein

**Affiliations:** 1 Department of Pharmaceutics & Industrial Pharmacy, Faculty of Pharmacy, Beni-Suef University, Beni-Suef 62521, Egypt; 2 Department of Clinical Pharmacy, Faculty of Pharmacy, Beni-Suef University, Beni-Suef 62521, Egypt; 3 Department of Clinical Pharmacy, College of Pharmacy, Al Ain University, Al Ain P.O. Box 112612, United Arab Emirates; 4 Department of Internal Medicine, Faculty of Medicine, Beni-Suef University, Beni-Suef 62521, Egypt; 5 Department of Medical Biochemistry and Molecular Biology, Faculty of Medicine, Badr University in Cairo, Cairo 11562, Egypt; 6 Department of Medical Biochemistry and Molecular Biology, Faculty of Medicine, Cairo University, Giza 12613, Egypt; 7 Department of Pharmaceutical Sciences, College of Pharmacy, Al Ain University, Al Ain P.O. Box 112612, United Arab Emirates; 8 Department of Pharmacology and Toxicology, Faculty of Pharmacy, Nahda University, Beni Suef 62511, Egypt; 9 Department of Clinical Pharmacy, Faculty of Pharmacy, October 6 University, Giza 12585, Egypt

**Keywords:** nanomedicine, gold nanoparticles, intra-articular, transdermal, effectiveness, bioavailability, inflamed joints

## Abstract

Nanomedicine has emerged as an important approach for targeting RA medication. Rheumatoid arthritis (RA) is a widespread autoimmune disorder marked by multiple inflamed joints. Gold nanoparticles (GNPs) have been demonstrated as efficacious nanocarriers due to their unique characteristics and the relative simplicity of their synthesis in varied sizes; moreover, they have the capability to alleviate several inflammatory markers. The current objective was to combine methotrexate (MTX) with GNPs to overcome MTX restrictions. GNPs were fabricated by a chemical reduction technique, utilizing sodium citrate and tween 20. The MTX-GNPs formulations were characterized in vitro by % entrapment efficiency (%EE), particle size, polydispersity index (PDI) zeta potential, and % release. The MTX-GNPs formulation was administrated as an intra-articular solution, and additionally, incorporated into a Carbopol gel to investigate its anti-arthritic effectiveness and bioavailability in vivo. The results indicated that a %EE of 87.53 ± 1.10%, and a particle size of 60.62 ± 2.41 nm with a PDI of 0.31 ± 0.03, and a zeta potential of −27.80 ± 0.36 mV were optimal. The in vitro release of MTX from the MTX-GNPs formulation demonstrated that the MTX-GNPs formulation’s release was 34.91 ± 1.96% and considerably (*p* < 0.05) lower than that of free MTX, showing a significant difference in dissolution patterns (*p* < 0.05). In vivo, MTX-GNPs formulations inhibited IL-6 by 36.52%, ACCP (63.25 %), COMP (28.16%), and RANKL (63.67%), as well as elevated IL-10 by 190.18%. Transdermal MTX-GNPs decreased IL-6 by 22.52%, ACCP (56.63%), COMP (52.64%), and RANKL (79.5%), as well as increased IL-10 by 168.37%. Histological investigation supported these recent findings. Conclusions: Marked improvements in MTX anti-arthritic effects are seen when it is conjugated to GNPs.

## 1. Introduction

Rheumatoid arthritis (RA) is a chronic inflammatory illness characterized by persistent high grade systemic inflammation, significant synovitis resulting in articular cartilage erosion, and is evidenced by pain, morning stiffness, and markedly restricted mobility [1]. A prior study supported the World Health Organization’s 1994 publication which found that rheumatic patients had a higher risk of developing osteoporosis and osteopenia [2]. Furthermore, RA patients are at high risk for the development of cardiovascular complications considering the recent 2019 WHO and the 2013 American College of Cardiology and American Heart Association’s (ACC/AHA) published reports [3]. Although the triggering cause of RA is unclear, it is assumed to be a product of genetic and environmental factors. Genetically, a specific epitome type in HLA-DRB1 alleles (SE) has been related to an increased likelihood of RA occurrence. Moreover, the most reported environmental triggering factor for RA is cigarette smoking. Hence, people with positive anti-citrullinated protein antibody (ACCP) are predisposed to RA due to the interaction between genetics and smoking. Furthermore, an additional triggering factor is gut microbiome change. The presence of ACCP with its two subsets is valuable in the early diagnosis of RA. Although the positive ACCP subset of RA is more aggressive than the negative ACCP subset, the negative ACCP subset is less responsive to MTX [4]. In addition, RA may have a noticeable impact on the mortality and morbidity of its patients, as one of its reported serious extra-articular manifestations is interstitial lung disease (RA-ILD). Even so, a specialized care plan for these cases has not yet been developed; consequently, it is essential to comprehend the pathogenesis as well as the clinical characteristics in order to better control and enhance a patient’s prognosis. Taking this into consideration, in order to enhance an RA-ILD patient’s prognosis, it is crucial to prevent and control the leading causes of death in situations of RA-ILD, which are lung cancer and infectious pneumonia [5]. The global prevalence of RA varies, with a higher incidence in industrialized nations; this may be due to exposure to environmental triggering factors. Around the world, women are more likely to have RA than men, with a prevalence ratio that ranges from 4:1 in young adults to less than 2:1 in geriatric populations with the disease [6]. Methotrexate is considered an important member of the disease-modifying anti-rheumatic medication class (DMARD) of medicines; it acts mainly as an antineoplastic and immuno-suppressive folic acid antagonist. It has been demonstrated that dihydrofolate reductase (DHFR) plays an important role in the synthesis of thymidylate; as a result, MTX inhibits its competitive activity, resulting in a decrease in purine and pyrimidine synthesis, which reduces cell proliferation, particularly in immune cells such as T lymphocytes, and this decreases the inflammation [7].

Although MTX in low doses is proven to be the first line agent in treatment plans among DMARDs in patients with RA, it has various side effects. Traditional routes of administration cause mucosal ulceration, stomach arthritis, suppression of bone marrow, hepatic fibrosis, and cirrhosis [8]. Additionally, currently marketed topical MTX formulations have poor penetration due to their hydrophilicity and their dissociation at physiological pH; however, a novel nanogel formed from an MTX-coupled nanostructured lipid carrier (MTX-NLC) has shown significant efficacy for topical MTX used in the treatment of psoriasis [9].

Developing novel drug delivery technologies for MTX is an important step for future research concerning the treatment of a variety of disorders through multiple modes of administration. A novel delivery system could involve a controlled burst release and introduce new administration routes [10]. Not only is the introduction of a novel delivery approach very important, but so is the route of administration of the drug because the intra-articular (IA) route of administration, directly at the joints, is a promising method for reducing undesirable systemic side effects [11].

GNPs are currently considered to be one of the most promising choices for delivering a variety of payloads to their intended destinations. The advantage of GNPs is their ease of fabrication and synthesis, as they are inert and non-toxic. Additionally, the latest systems of drug delivery utilizing GNPs improve the solubility of the free drug, as well as the drug’s in vivo stability and bio-distribution. This study aimed to estimate and compare the modulatory effects of coupling MTX to GNPs with MTX as a free drug [12].

## 2. Results

### 2.1. In Vitro Evaluation of MTX-GNPs Formulation

To quantify MTX, a spectrophotometric technique in the UV/Vis range was devised. At a wavelength of 303 nm, the MTX calibration curve was obtained spectrophotometrically (JascoV530, Easton, MD, USA). The method of quantitative measurement of MTX was reliable and accurate because there was a linearity in the relationship between MTX concentration and absorbance; this relationship follows Beer Lambert law with an R2 of 0.999 as the coefficient of determination. The percentage of EE was used to calculate the drug content of the manufactured formulations. EE was determined to be 87.53 ± 1.10 percent.

Dynamic light scattering results indicated a vesicle distribution that was homogeneous and had a low polydispersity index. The vesicle diameter or size was measured to be 60.62 ± 2.41 nm, with a PDI of 0.31 ± 0.03. To estimate the surface charge of NPs, the zeta potential approach was used. In addition, the zeta potential can be used to forecast the colloidal stability of the final formulation. The result of zeta potential was −27.80 ± 0.36 mV. The zeta potential values of the MTX-GNPs formulation suggested a sufficiently negative surface charge for electrostatic stability.

**Figure 1:**
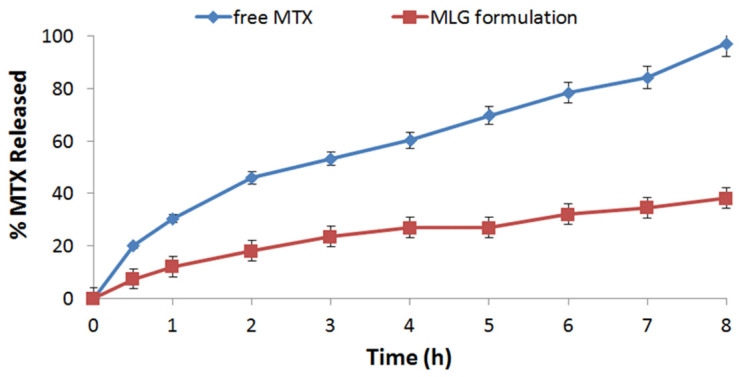
In vitro release profile of MXT from free MXT and MLG formulations (*n* = 3 ± SD).

The release of both free MTX and the MTX-GNPs formulation were evaluated and measured. Figure 1 revealed that the MTX-GNPs formulation’s release was considerably (*p* < 0.05) lower than that of free MTX. In vitro release kinetics and the mechanism of MTX from the MTX-GNPs formulation were evaluated through relaying on the DDSolver program. The Higuchi model was chosen to fit the data of release results due to its maximum R2 (0.9922), least AIC (24.3587), and MSC (3.578), as shown in Table 1. The Fickian diffusion mechanism was the mechanism of the release of MTX from the MTX-GNPs formulation because of the calculated “n” recorded as 0.417 ± 0.01. The similarity factor f2 was also calculated [13], and it was 34.91 ± 1.96%, showing a significant difference in dissolution patterns (*p* < 0.05).

**Figure 2:**
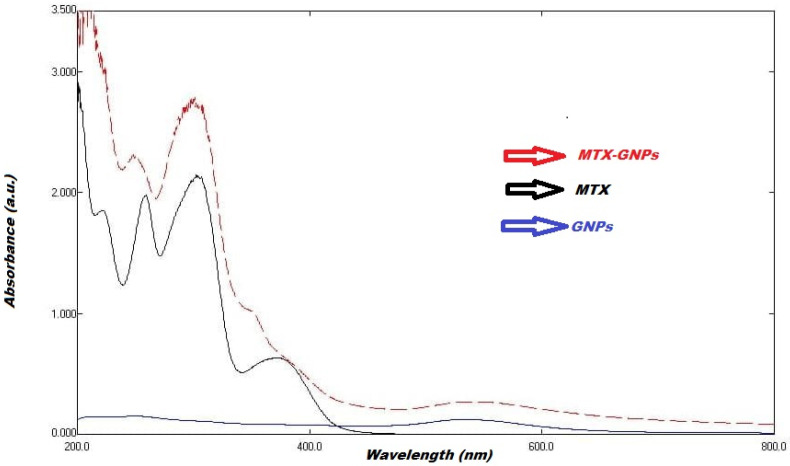
Comparative UV-Vis absorption spectra of free MTX, GNPs, and MTX-GNPs.

The optical features of the prepared GNPs and GNPs-MTX conjugates, measured by UV-Vis spectroscopy, were depicted in Figure 2. Free MTX exhibited three characteristics. Absorption peaks were centered at 258, 306 and 372 nm. Using UV-visible spectroscopy with a VWR UV-1600PC UV/VIS spectrophotometer, samples were analyzed in the 400–900 nm spectral range. The crystalline structure of GNPs was examined using a BRUKER D8 ADVANCE diffractometer (Kα Cu) with a LINEXE detector.

**Figure 3:**
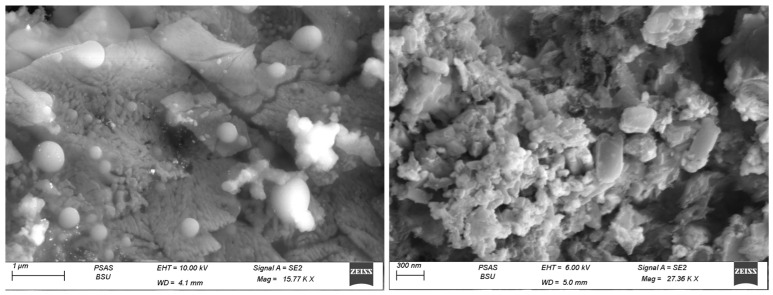
Surface morphology of MTX-GNPs by SEM.

The morphology of MTX- GNPs was examined by SEM analysis. Images in Figure 3 confirmed the formation of homogeneous size, mostly spherical, and highly dispersed GNPs. SEM measurements were examined utilizing the Scanning Electron Microscope (SEM, Carl Zeiss, Jena, Germany). The formulation was adhered to the surface of the carbon-coated copper grid, and the film was observed under SEM at suitable magnifications.

### 2.2. In Vivo Evaluation of MTX-GNPs Formulation

**Figure 4:**
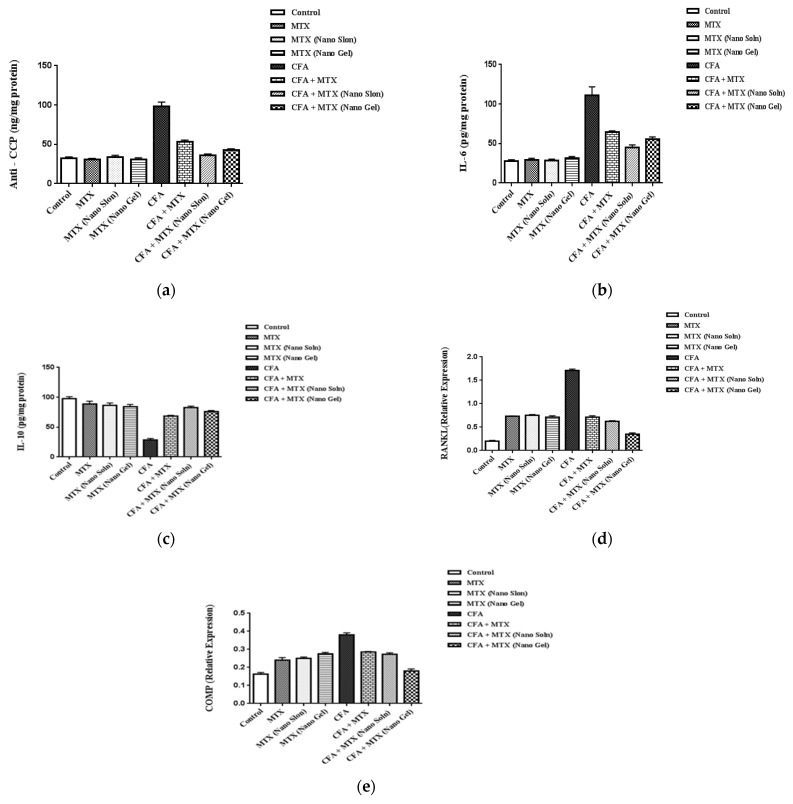

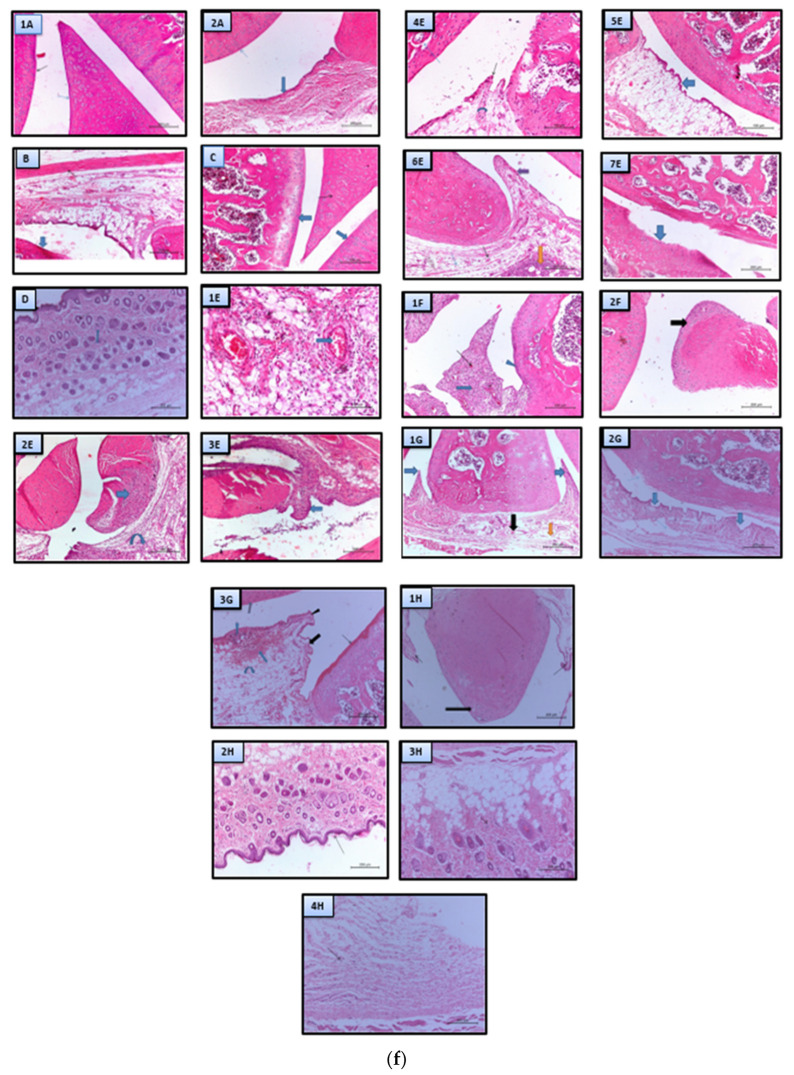
(**a**) Effect of MTX, MTX-Nano solution, and MTX-Nano gel on Anti-CCP in CFA induced rheumatoid arthritis; (**b**) effect of MTX, MTX-Nano solution, and MTX-Nano gel on IL6 in CFA induced rheumatoid arthritis; (**c**) effect of MTX, MTX-Nano solution, and MTX-Nano gel on IL-10 in CFA induced rheumatoid arthritis; (**d**) effect of MTX, MTX-Nano solution, and MTX-Nano gel on RANKL in CFA induced rheumatoid arthritis; (**e**) effect of MTX, MTX-Nano solution, and MTX-Nano gel on COMP in CFA induced rheumatoid arthritis; (**f**) histopathologic examinations under a light microscope on knee joints by H&E staining (magnification ×200 and ×100).

The effects of MTX, MTX-Nano solution, and MTX-Nano gel on Anti-CCP in CFA induced rheumatoid arthritis were investigated. As shown in Figure 4a, we observed that the CFA-arthritis group had a noticeable increase in plasma ACCP level compared to the normal control group. However, our manuscript revealed that the CFA + MTX group had a marked inhibition of serum ACCP by 45.58% compared to the CFA-arthritis group. In addition, the CFA + MTX-GNPs group reported remarkable inhibition of serum ACCP by 63.25%, when compared to the CFA-arthritis group. Moreover, the CFA + MTX-GNPs gel formula group documented inhibition of serum ACCP by 56.36%, compared to the CFA-arthritis group.

The effects of MTX, MTX-Nano solution, and MTX-Nano gel on IL-6 and IL-10 in CFA induced rheumatoid arthritis were represented in Figure 4b. Our results demonstrated that the CFA-arthritis group recorded a significant increase in plasma IL-6 level compared to the normal control group. However, our investigations discovered that the CFA + MTX group displayed significant inhibition of serum IL-6 by 9.79%. Moreover, the CFA + MTX-GNPs group showed significant inhibition of serum IL-6 by 36.52% when compared to the CFA-arthritis group. Additionally, the CFA + MTX-GNPs gel formula group experienced significant inhibition of serum IL-6 by 22.52% compared to the CFA-arthritis group. As noticed in Figure 4, the CFA-arthritis group exhibited a significant decrease in plasma IL-10 level compared to the normal control. Moreover, our results revealed that the CFA + MTX group achieved marked elevation of serum IL-10 by 139.43%. In addition, the CFA + MTX-GNPs group showed a noticeable elevation of serum IL10 by 190.18% compared to the CFA-arthritis group. In addition, the CFA + MTX-GNP gel formula group registered a significant increase in serum IL-10, by 168.37%, compared to the CFA-arthritis group.

The effects of MTX, MTX-Nano solution, and MTX-Nano gel on COMP and RANKL in CFA induced rheumatoid arthritis were shown in Figure 4d. This study demonstrated that the CFA-arthritis group exhibited marked elevation in their COMP levels compared to the normal control group. However, our review confirmed that the CFA + MTX group revealed marked COMP inhibition of 25%. In addition, the CFA + MTX-GNPs group reported significant inhibition of COMP by 28.16% when compared to the CFA-arthritis group. In addition, the CFA + MTX-GNPs gel formula group displayed significant inhibition of COMP by 52.64% compared to the CFA-arthritis group. As shown in Figure 4e, it has been noticed that the CFA-arthritis group exhibited a pronounced increase in RANKL level compared to the normal control group. However, this manuscript identified that the CFA + MTX group reported remarkable inhibition of RANKL by 58.42%. As well as this, the CFA + MTX-GNPs group documented significant inhibition of RANKL by 63.67% when compared to the CFA-arthritis group. Additionally, the CFA + MTX-GNPs gel formula group displayed remarkable inhibition of RANKL by 79.5% compared to the CFA-arthritis group.

The effects of MTX, MTX-Nano solution, and MTX-Nano gel on histopathological changes were measured. Histopathologic examinations under a light microscope on rats’ knee joints were conducted by routine H&E staining (magnification ×200 and ×100), as shown in Figure 4f. We demonstrated the following findings. 1A: Sections from normal control rats showed a normal articular surface (black arrow) with a normal meniscus (blue arrow). 2A: Knee joint capsule sections showed normal joint articular surface (small arrow) with the normal synovial membrane epithelium (large arrow) that the normal control group showed. 1E: Sections from the CFA-group showed congestion of the vasculature (blue arrow) together with infiltration of inflammatory cells and edema (black arrows). 2E: There was partial organization of the meniscus (blue arrow) together with edema and inflammatory cells at the joint capsule (bent arrow). 3E: Proliferation of the synovial membrane (blue arrow). 4E: Dentation of the synovial membrane (black arrow) edema in the joint capsule (bended arrow). 5E: Proliferation and dentation of the synovial membrane (blue arrow). 6E: Congestion (black), oedema (blue), and lymphocytic aggregation (orange) together with a prolapse of the synovial membrane (violet). 7E: A focal eroded articular surface (blue). 1F: Sections from the CFA + MTX group showed congestion of the vasculature (black) together with mononuclear cell infiltration of the joint capsule (blue), and also proliferation of the articular surface (arrowhead). 2F: Projection of the synovial membrane (blue), congestion (black), and edema (orange). 1G: Sections from the CFA + MTX (Nano Soln) group showing prolapse of the synovial membrane (blue arrow), congestion (black arrow), and edema (orange arrow). 2G: Dentation of synovial membrane (thick arrow) together with edema in the joint capsule (thin arrow), tea red articular surface (thin arrow). 3G: The joint capsule showed edema (bended arrow), congestion (blue arrow), and focal aggregation of inflammatory cells (arrowhead); in addition, prolapse (black arrowhead) and dentation (black arrow) of the synovial membrane could be observed; the articular surface was either normal (orange arrow) or hyper plastic (thin arrow). 1H: Sections from the CFA + MTX (Nano Gel) group showed a prolapsed synovial membrane (thin arrow), and mild partial organization of the meniscus (thick arrow). 2H: Skin showed slight hyperkeratosis (thin arrow). 3H: Dermal skin showed proliferation of the fibrous connective tissue in the deep dermis (arrow). 4H: Dermal skin showed lymphocytic infiltration in the derma (arrow).

(a) Each value represents the mean of six experimental rats ± SEM. Statistical analysis was performed using one-way ANOVA followed by the Student–Newman–Keuls multiple comparisons test. a = significantly different from control group at *p* < 0.05; b = significantly different from MTX group at *p* < 0.05; c = significantly different from MTX (Nano Soln) group at *p* < 0.05; d = significantly different from MTX (Nano Gel) group at *p* < 0.05; e = significantly different from CFA group at *p* < 0.05; f = significantly different from CFA + MTX group at *p* < 0.05.
(b) Each value represents the mean of six experimental rats ± SEM. Statistical analysis was performed using one-way ANOVA followed by Student–Newman–Keuls multiple comparisons test. a = significantly different from control group at *p* < 0.05; b = significantly different from MTX group at *p* < 0.05; c = significantly different from MTX (Nano Soln) group at *p* < 0.05; d = significantly different from MTX (Nano Gel) group at *p* < 0.05; e = significantly different from CFA group at *p* < 0.05; f = significantly different from CFA + MTX group at *p* < 0.05.
(c) Each value represents the mean of six experimental rats ± SEM. Statistical analysis was performed using one-way ANOVA followed by Student–Newman–Keuls multiple comparisons test. a = significantly different from control group at *p* < 0.05; b = significantly different from MTX group at *p* < 0.05; c = significantly different from MTX (Nano Soln) group at *p* < 0.05; d = significantly different from MTX (Nano Gel) group at *p* < 0.05; e = significantly different from CFA group at *p* < 0.05; f = significantly different from CFA + MTX group at *p* < 0.05.
(d) Each value represents the mean of six experimental rats ± SEM. Statistical analysis was performed using one-way ANOVA followed by Student–Newman–Keuls multiple comparisons test. a = significantly different from control group at *p* < 0.05; b = significantly different from MTX group at *p* < 0.05; c = significantly different from MTX (Nano Soln) group at *p* < 0.05; d = significantly different from MTX (Nano Gel) group at *p* < 0.05; e = significantly different from CFA group at *p* < 0.05; f = significantly different from CFA + MTX group at *p* < 0.05; g = significantly different from CFA + MTX (Nano Gel) group at *p* < 0.05.
(e) Each value represents the mean of six experimental rats ± SEM. Statistical analysis was performed using one-way ANOVA followed by Student–Newman–Keuls multiple comparisons test. a = significantly different from control group at *p* < 0.05; b = significantly different from MTX group at *p* < 0.05; c = significantly different from MTX (Nano Soln) group at *p* < 0.05; d = significantly different from MTX (Nano Gel) group at *p* < 0.05; e = significantly different from CFA group at *p* < 0.05; f = significantly different from CFA + MTX group at *p* < 0.05.
(f) Histopathologic examinations under a light microscope on knee joints by H&E staining (magnification ×200 and ×100).

## 3. Discussion

Pathogenesis and optimal treatment therapeutic approaches for RA have notably advanced during the past decade. Both biological and conventional therapies are currently accessible to effectively control RA, and are considered the important therapeutic approaches and treatment strategies [14]. For most RA patients, MTX is suggested to be the current gold standard that treats a wide range of autoimmune diseases and other disorders including some types of cancer; however, the medication’s poor pharmacokinetic profile and tight safety margin have hampered the therapeutic outcomes of traditional drug administration systems. As a result of these limitations of traditional administration routes, MTX has been used and tried IA in clinical trials, considering the advantages of delivering high drug concentrations selectively to their site of action using a low dose to reduce overall undesirable side effects. However, because IA MTX administration may result in rapid diffusion and efflux of the drug from the synovium and joint cavity into the bloodstream, frequent IA injection may be required [15]. Hence, scientific research turned to reducing this limitation by introducing innovative agents in MTX delivery.

Relying on these findings, as well as the fact that MTX is a basic systemic therapy for many diseases—and to overcome the drawbacks and limitations of MTX, boost its anti-inflammatory activity, penetrability, permeation, bioavailability, and minimize its side effects—motivated us to search and look into different pharmaceutical approaches to see whether the intra-articular injected and topical MTX-GNPs formulations would be more effective in vivo and in vitro than MTX on its own. In this work, the linking of MTX to GNPs was determined in order to improve MTX penetrability, bioavailability, and effectiveness.

Previous research attempts reported effective targeting of MTX via different nanocarrier moieties. MTX was successfully loaded onto stealth liposomes via a thin-film hydration method. The prepared nanolipid vesicles optimized MTX targeting in vivo, and efficaciously enhanced MTX retention in the targeted tissue in comparison to MTX on its own [16]. Moreover, conjugation of MTX to poloxamer by esterification is a novel strategy (p–MTX micelles) that has led to improvements in MTX pharmacokinetics [17].

Gold nanoparticles have been presented for a variety of applications in diverse fields and they are especially appealing because their valued size, as well as shape-dependent properties, are simple to control and modify [12]. In light of this, a prospective MTX-GNPs formula has been prepared to encapsulate MTX, in order to achieve better drug permeation and bioavailability. In addition, new topically applied methodologies are being investigated [18]. In general, linking MTX to GNPs aids in more effective and selective uptake, a property distinct to nanocarriers, which improves therapeutic potential and lowers effective doses [19]. As a result, it should be included in GNPs to boost its anti-inflammatory properties.

GNPs are composed of gold III, sodium citrate, and tween 20 [20]. Initial trials were conducted to determine and identify the concentration ranges of gold III, tween 20, and sodium citrate, as control variables. Before obtaining the final concentration ranges that showed up in the article, preliminary tests were carried out to select the appropriate independents. To choose the best MTX-GNPs preparation formulation, optimization was considered. The MTX-GNPs formulation of choice was composed of gold III (0.01 gm) chloride, sodium citrate (0.015 g), and (0.025 g) of tween 20. These observations were supported by various studies [19,20,21]. MTX-GNPs formulation was successfully synthesized and prepared. The concentration of the drug in the prepared formula was calculated using entrapment efficiency (EE%). Tween 20 is a surfactant that aids in increasing and enhancing the solubility of poorly aqueous drugs [22]. Preliminary attempts demonstrated that presence of sodium citrate has a statistically antagonistic effect on EE%, example (f1, f11). As presented in Table 2, f1 has an EE% of 47.42 ± 2.10%, but f11 has an EE% of 68.76 ± 2.15%. Moreover, preliminary tests revealed that GNPs have statistically synergistic effects on EE%, as confirmed in f2, f5. Of which, f2 has an EE% of 80.32 ± 0.42, but f5 has an EE% of 48.31 ± 1.18. In addition, preliminary trials confirmed the synergistic effect of tween on EE%, as shown by f2, f10. F2 has an EE% of 80.32 ± 0.42, f10 has an EE% of 75.89 ± 4.40. Finally, preliminary data confirmed that all variables were inversely proportional to release percentage, demonstrating successful sustained release.

Increasing MTX dissolution rate contributed to increased drug loading and improved MTX entrapment efficiency. The results of particle size estimation indicated that MTX-GNPs formulation was fabricated with small particle size, narrow, and limited PDI. This PDI illustrated a relatively homogenous distribution of particle size which also helped to improve the physicochemical properties of the generated NPs. It also enabled effective transdermal delivery of active ingredients. Furthermore, the MTX-GNPs formulation was synthesized with a negative zeta potential, revealing that the colloidal system may be stable because electrostatic repulsion between particles restricts coalescence, and therefore stabilizes the nano particulate diffusion and dispersion [23]. Additionally, MTX-GNPs exhibited improved drug release within 8 h, according to an in vitro drug release study, since the presence of small particles inhibited and reduced the mean particle size of the resulting particles and expanded particle surface area, which improved MTX release [20,21]. The DDSolver software was utilized to assess the in vitro release kinetics and pathway of MTX release from the MTX-GNPs formulation. The Higuchi model predicted that MTX would be released from the MTX-GNPs formulation with a release scale factor “n” of 0.417, implying that Fickian diffusion would be used.

According to a preceding in vivo experimental study, we utilized CFA to develop an aggressive modified arthritis model on Wistar Albino rats [24], and our in vivo study confirmed and approved that the induction of arthritis was associated with elevation of levels of ACCP, IL-6, COMP and RANKL, along with inhibition of IL-10 level. Our study indicated that injection of CFA, in rats, resulted in marked inflammation, indicated by a significant increase in serum IL-6, along with a notable decrease in serum IL-10. These inflammatory reactions were also reported by [25] who illustrated that CFA induced rats’ arthritis, showing an increased level of IL-6 and a reduced level of IL-10.

Moreover, our results illustrated that rats IA injected by MTX-GNPs solution, compared to rats treated with IA MTX on its own, showed significantly decreased serum levels of IL-6 and increased serum IL-10. These results confirmed our investigations that applying GNPs to IA delivery was a potential technique for improving treatment efficacy and compliance in patients with RA [26], where GNPs appeared to block production of proinflammatory IL-6 cytokines in Jurkat and U937 cells due to a decrease in phosphorylation of extracellular signal-regulated kinases (ERK). In addition, previous results illustrated that coupling with GNPs could add an immunomodulatory function to the conjugated drug by impairing the production of pro-inflammatory cytokines [27]. Moreover, a previous review had shown an increase in anti-inflammatory cytokine IL-10 secretion from macrophages, in a dose dependent manner of GNPs, in the hepatic injury rat model [28].

Additionally, our study revealed that rats treated with transdermal MTX-GNP gel formula exhibited a marked decrease in the serum level of IL-6 along with increased serum IL-10, compared to rats treated with intra-articular MTX on their own. Previous research confirmed our documented results suggesting that conjugation of MTX to GNPs could improve its transdermal application, in which the drug could be seen both in the epidermis and, to a lesser extent, in the dermis. On the contrary, samples from rats administered an MTX-only-based formulation demonstrated an MTX peak in the epidermis, while it was nonexistent in the dermis, suggesting greater drug absorption through the skin by using the GNPs as a drug carrier [29]. However, our results demonstrated that IA MTX-GNPs formula exhibited significant decrease in serum level of IL-6 and increased serum IL-10, more than the transdermal MTX-GNP gel formula. Previous studies recommended IA delivery of MTX to be more efficient than topical administration [30].

Our study indicated that injection of CFA in rats resulted in marked bone and cartilage destruction, indicated by a significant increase in RANKL and COMP level. These inflammatory reactions were also reported by [25], who illustrated that CFA induced rats’ arthritis and showed an increase in RANKL level. Other results indicated that induction of RA was associated with COMP elevation [31]. Moreover, our study illustrated that rats who were intra-articularly injected with MTX-GNPs solution, compared to rats treated with IA MTX on its own, showed significantly decreased levels of RANKL and COMP. These results confirmed our assumptions that applying GNPs to IA delivery of MTX reduced inflammation and bone erosion in RA by attenuating NF-κB signaling [32]. A similar phenomenon was that GNPs dramatically reduced NF-B signaling-mediated inflammation in diabetic rats’ livers or in liver injury, consequently inactivating or repressing liver macrophages in order to induce hepatocellular apoptosis [33]. In addition, GNPs might block the RANKL induced osteoclast formation leading to cartilage and bone destruction [34].

Our results confirmed a notable decrease in COMP serum level in rats that were intra-articularly injected with MTX-GNPs solution, compared to rats treated with IA MTX on its own. This was in line with a previous study demonstrating that GNPs could protect cartilage tissue in arthritis with mechanisms other than COMP, inhibit angiogenic activity (by binding vascular endothelial growth factor (VEGF) and inhibiting the proliferation and migration of endothelial cells), or act as an antioxidant (inhibit reactive oxygen species (ROS) and prevent the destruction of RA synovitis) [35]. So, it is important to note that this study represented one of the initial initiatives for investigating the protective effect of GNPs on cartilage tissue by measuring COMP level. Furthermore, this manuscript demonstrated that rats, treated with transdermal MTX-GNP gel formula, exhibited a marked decrease in serum level of RANKL and COMP compared to rats treated with IA MTX-GNPs or IA MTX on its own. COMP is an inflammatory related marker for cartilage complication as well as MMP. Moreover, the concentration of COMP in synovial fluid was reported to be ten times greater than in serum [36]. A previous study confirmed novel attempts in nano-medication, depending on inflammation-related enzymes which were abundant significantly in inflamed joints, resulting in selective release of medication in the targeted region [37].

Finally, our study indicated that injection of CFA in rats resulted in marked inflammation, indicated by a significant increase in serum ACCP. These inflammatory reactions were also reported by [24], who illustrated that CFA induced rats’ arthritis and resulted in an increase in serum ACCP level. Moreover, our study illustrated that rats who were intra-articularly injected with MTX-GNPs solution, compared to rats treated with IA MTX on its own, exhibited a significantly decreased serum level of ACCP. Additionally, rats treated with transdermal MTX-GNP gel formula exhibited a marked decrease in serum level of ACCP, compared to rats treated with IA MTX on their own. Our result attributed that to the potential anti-arthritic effect of GNPs. From another perspective, our investigations demonstrated that IA MTX-GNPs formula exhibited a significant decrease in serum level of ACCP, more than that of the transdermal MTX-GNP gel formula; we attributed this to the preferable systemic absorption of MTX IA, rather than transdermal. It is crucial to remember that this study is one of the first attempts to investigate the effect of conjugation of GNPs to MTX on the ACCP level. Our histopathological study supported our findings in this current work.

As a result, we can deduce that this study provided empirical proof that such a delivery system provides a prolonged plasma profile of MTX, and augmented the drug’s specific activity in vitro and in vivo in animal models. It is important to take into consideration that our study is one of the initial initiatives to boost IA and topical delivery effectiveness of MTX with the help of nanostructured delivery. Additionally, we measure the effect of loading MTX to GNPs on new distinguishable markers, such as COMP and ACCP.

In comparison with our findings, recent research reported that MTX loaded multifunctional nanoparticles such as poly(ethylene glycol)-poly(lactic-co-glycolic acid) (PLGA) NPs may be a novel therapeutic approach for the treatment of RA [38]. This study presented a type of photothermal nanoparticle that was designed to selectively release the medication in the photothermally heated affected region, after exposure to localized near infrared (NIR) light. On the other hand, our study investigated a distinguishable approach depending on pH-sensitive nanoparticles, which were designed to effectively release medications in targeted joints by utilizing the pH differential pattern between normal and inflamed regions. The pH values in inflammatory tissues, such as inflamed joints, are remarkably low, varying between 6.6 and 6.8 [39]. The most apparent advantage of our study over previously mentioned studies was the utilization of a simple procedure, in contrast to the majority of developed studies that usually generated complicated multiple-step methods. Regarding the development of RA nanomedicine for clinical uses, manufacturing techniques and budgets for large-scale production must be carefully considered. In addition, our attempt is promising because it is applicable in many fields. However, NIR light’s poor penetration depth limits its practical applicability in cancer treatments. Finally, introducing intra-articular delivery to nanomedicine allows for more efficient localization of the drug in the inflamed joint. Another piece of research utilized GNPs to target MTX as (GNPs/MTX-Cys-FA) via a series of three step processes [40]. In vitro characterizations of our generated MTX-GNPs formulation were proven to be more preferable than (GNPs/MTX-Cys-FA). Moreover, our study investigated a variety of immunological markers in vivo, including serum markers (ACCP, IL-6, IL10) and tissue markers (RANKL, COMP). In addition, we utilized different assay techniques (ELISA, Western Blot, PCR) in the evaluation of these markers. 

Furthermore, attaching GNPs to the surface of two-dimensional nanomica platelets (NMPs) resulted in successful fabrication of a flexible hybrid substrate, utilized in biosensors, promoting fast and accurate bio-detection of both hydrophilic and hydrophobic bacteria via surface-enhanced Raman scattering (SERS) [41]. In addition, potential anti-malaria medication was developed using glucose-based ultra-small gold nanoparticles or gold nanoclusters (Glc-NCs), due to effective binding of (Glc-NCs) extracellularly, as well as intra-erythrocytic phases of plasmodium falciparum (which is the most severe and common protozoa causing malaria illness). Moreover, (Glc-NCs) improved water solubility of ciprofloxacin as an antibiotic with limited water solubility, and little antimalarial activity [42]. In addition, indole derivative-capped gold nanoparticles (Au IDs) were generated and demonstrated potent antibacterial activity against both laboratory antibiotic-sensitive as well as multidrug-resistant (MDR) bacteria. Alongside that, Au IDs conclusively proved exceptional potential in MDR bacterial wound infections [43]. Finally, betulinic acid (BA)-functionalized GNP exhibited selective cytotoxicity as well as stronger antiproliferative effects, particularly in comparison to BA alone. These nanoconjugates effectively suppressed mitochondrial respiration, confirming their mitocan activity [44].

Reviews of the literature indicated that invasomes were effective drug delivery systems that increased transdermal flux through deep skin layers. Invasomes are novel transdermal methods that have been investigated to increase drug absorption, bioavailability, and therefore drug activity, and are composed of ethanol, terpenes, phospholipids, and cholesterol. Phospholipids serve as the basis for lipid bilayers [13]. Hence, we look forward, in future, to achieving more MTX transdermal penetrability and bioavailability through lipid nanocarrier types.

### 3.1. Materials

Gold (III) chloride, methanol, and tween 20 were acquired from Thermo-Fisher Inc (Pittsburgh, PA, USA). Methotrexate, sodium citrate, and complete Freund’s adjuvant (CFA) were purchased from Sigma-Aldrich Chemical (St. Louis, MO, USA). Quantitative Enzyme-Linked Immunosorbent Assay (ELISA) kits for serum IL-6 and IL-10 were purchased from R&D Systems Europe Company (Abingdon, UK, 19 Barton LaneAbingdon, Science Park Abingdon, Europe). Anti-cyclic citrullinated peptide antibody (ACCPAb) ELISA kits were obtained from My BioSource Company (San Diego, CA, USA). Thermo Fisher Scientific Company (168 Third Avenue, Waltham, MA, USA 02451) was the provider of Polyclonal antibodies against RANKL for Western Blot analysis (Waltham, MA, USA). Bio BASIC Company provided the radio-immunoprecipitation assay (RIBA) lysis buffer PL005, and the Bradford protein assay kit utilized for quantitative protein analysis (Marhham, ON, Canada). TGX Stain-Free™ FastCast™ acrylamide kit [sodium dodecyl sulfate-poly acrylamide gel electrophoresis (SDS-PAGE)] was provided by Bio-Rad Laboratories (Hercules, CA, USA). Thermo Fisher Scientific Company also provided high-capacity cDNA reverse transcription kit ferments and primer for real-time polymerase chain reaction (RT-PCR) investigation of COMP (Waltham, MA, USA). Qiagen tissue extraction kit was used to isolate total RNA (Manchester, UK). All secondary antibodies used were obtained from Sigma-Aldrich Chemical Company (St. Louis, MO, USA).

### 3.2. Preparation of Methotrexate-Loaded Gold Nanoparticle Formulation

The methotrexate loaded on gold NPs (GNPs) MTX-GNPs formulation was generated by a chemical reduction method [20]. The chemical reduction method appears to be a simple procedure that only necessitates the reagents to be mixed under well-defined external circumstances. These circumstances can have a slight effect on the final morphology of the nanoparticle. As shown in Table 1, we added gold III (0.01 gm) chloride solution into 100 mL of phosphate buffer and constantly heated it to boiling. We added sodium citrate (0.015 gm) to the boiling gold solution, and the reducing agent solution was added drop by drop with continuous stirring for 1 min. The color of HAuCl4 solution changed from pale yellow to dark red over several minutes. The stirring process was continued for another 10 min for complete homogenization. Then, 0.025 gm of tween 20 was added with continuous stirring for 5 min until a brilliant-red color appeared. MTX (10 mg) was mixed with the prepared colloidal solution of GNPs with continuous stirring at room temperature. The resulting solution was centrifuged for 30 min at 15,000 rpm. Finally, the supernatant was kept at 4 °C for further use. Since the HAuCl_4_ is corrosive, a glass spatula was used to avoid contact with metal. In the preparation of GNPs, the cleaning of glassware is very crucial. Thus, all the glassware and stir magnetic bars were thoroughly cleaned in freshly prepared aqua regia (HCl/HNO3 3:1, *v*/*v*), and then rinsed with distilled water and dried to avoid aggregation of residual gold particles and to avoid unwanted nucleation during synthesis. The liquid was vigorously stirred by Teflon-coated magnetic bars. No refluxing was utilized, in order to prevent the presence of temperature gradients in the liquid.

**Table 1:** DDSolver release data kinetic models to assess the release behavior of MXT from MLG formulation.

Release Data Models	Parameters of Goodness of Fit		
	R2	MSC	AIC
Zero-order	0.7873	0.9968	47.5961
First-order	0.8398	1.2805	45.0428
**Higuchi**	**0.9922**	**3.578**	**24.3587**
Korsmeyer-Peppas	0.9844	3.388	26.0736
Hixson-Crowell	0.8209	1.1671	45.8496
Hopfenberg	0.8368	1.0376	47.0152
Quadratic	0.8974	1.5014	42.8408
Baker-Lonsdale	0.9803	3.39	25.844

R2: Coefficient of determination; AIC: Akaike Information Criterion; MSC: Model Selection Criterion.

**Table 2:** Composition and in vitro characterization of MTX loaded gold nanoparticles.

Formula No	X1	X2	X3	Y1	Y2	Y3	Y4
F1	0.01	0.0025	0.0025	47.42 ± 2.10	60.99 ± 0.57	458.56 ± 35.42	0.612
F2	0.03	0.0016	0.002	80.32 ± 0.42	34.51 ± 0.49	369.1 ± 3.60	0.549
F3	0.02	0.00075	0.002	89.98 ± 1.03	35.83 ± 0.76	416.6 ± 8.83	0.585
F4	0.02	0.0016	0.0025	64.91 ± 2.67	47.80 ± 0.88	436.21 ± 20.17	0.410
F5	0.01	0.0016	0.002	48.31 ± 1.18	64.81 ± 1.02	381.63 ± 8.43	0.422
F6	0.03	0.0025	0.0025	61.35 ± 2.19	60.53 ± 0.87	144.4 ± 1.76	0.327
F7	0.02	0.0016	0.0025	64.52 ± 4.43	47.80 ± 0.96	391.2 ± 49.49	0.578
F8	0.01	0.0016	0.003	42.53 ± 1.04	27.03 ± 0.89	241.43 ± 8.0	0.364
F9	0.02	0.00075	0.003	89.52 ± 1.92	32.01 ± 0.58	863.23 ± 52.60	0.872
F10	0.03	0.0016	0.003	75.89 ± 4.40	50.64 ± 0.64	320 ± 5.67	0.354
F11	0.01	0.00075	0.0025	68.76 ± 2.15	32.20 ± 0.61	157.4 ± 19.51	0.356
F12	0.02	0.0025	0.002	61.09 ± 4.76	32.62 ± 0.74	861.1 ± 2.61	0.856
F13	0.02	0.0016	0.0025	63.96 ± 3.60	47.80 ± 0.86	391.2 ± 49.49	0.578
F14	0.03	0.00075	0.0025	92.05 ± 1.74	33.89 ± 0.77	315.2 ± 14.91	0.446
F15	0.02	0.0025	0.003	53.49 ± 0.92	49.97 ± 0.56	845.6 ± 2.45	0.823

X1 (gold concentration); X2 (sodium citrate concentration); X3 (tween); Y1 (EE%); Y2 (release%); Y3 (particle size); Y4 (PDI).

### 3.3. In Vitro Evaluation of MTX-GNP Formulation

#### 3.3.1. Measurement of % Entrapment Efficiency

Methotrexate content was determined by cooling centrifuging (SIGMA, Steinheim, Germany). A sample of the prepared MTX-GNPs formulations were centrifuged at 20,000 rpm for 1 h [45]. The supernatant solution was collected and the %EE was calculated in triplicates using the UV-VIS spectrophotometer method at the maximum wavelength of 303 nm as follows:

%EE = (B − A)/B × 100(1)

where A is the drug amount in supernatant, and B is the total drug amount.

#### 3.3.2. Evaluation of Particle Size, Polydispersity Index, and Zeta Potential

Dynamic Light Scattering (DLS) was applied to evaluate the polydispersity index (PDI), zeta potential, and particle size of MTX-GNPs (Zetasizer Nano ZN, Malvern An-alytical Ltd., Enigma Business Park, Grovewood Road, Malvern, WR14 1XZ, UK) [46]. At 25 °C, samples were tested after being diluted in ultra-purified water.

#### 3.3.3. In Vitro Release Studies

Samples of free MTX and MTX-GNPs were loaded on a dialysis bag as a diffusion membrane [47]. A total of 50 mL of phosphate buffer saline (PBS, pH 7.4), at 100 rpm and 37 ± 0.5 °C, was used as the dissolution medium. At predefined time points, samples of 1 mL were taken and replaced with an equal volume. MTX content was calculated using the UV-VIS spectrophotometer method at the maximum wavelength of 303 nm to measure the %MTX released in triplicate.

#### 3.3.4. Kinetic Analysis of Release Data

The DDSolver program software was used to determine the kinetics of MTX’s release [13], and we used the Korsmeyer–Peppas equation to identify the mechanism of the release. The DDSolver chose the model that best matches the MTX release data based on the criteria of obtaining the lowest Akaike information criterion (AIC), the highest coefficient of determination (R2), and the highest model selection criterion (MSC). The mechanism was Fickian diffusion if n = 0.5, and was non-Fickian diffusion if 0.5 < n < 1. Furthermore, the DDSolver calculated the similarity factor “f2” to determine the significance (*p* < 0.05) of the difference in dissolution profiles between MTX-GNPs formulation and free MTX. The difference was insignificant if f2 > 50, and was significant if f2 < 50.

### 3.4. In Vivo Characterization

#### 3.4.1. Animals

Forty-eight adult female Wistar Albino rats weighing 170–200 g were acquired from the animal house of the Faculty of Pharmacy, Nahda University. Rats were housed in circumstances that included controlled humidity (60%), temperature (22 ± 1 °C), and continual illumination (light–dark cycle of 12 h). Throughout the trial, animals were given unlimited access to a conventional rat chow diet and water. Before the trial, the animals were housed under the same circumstances for one week to acclimate. BSU-IACUC committee approval number (022-282).

#### 3.4.2. Experimental Design

The animals were randomly assigned to eight groups of rats after one week of acclimatization as follows:

Group 1: normal untreated control group, rats were just given saline and were not given any medicine.
Group 2: CFA-arthritic group, rats were given three subcutaneous doses of CFA on days 1, 5, and 9, each dose containing 0.4 mL into the right hind legs plantar surface.
Group 3: MTX-standard group, from day 14 to day 28, rats received MTX intra-articularly at a dose of 0.1 mg/kg/day (one dose per week).
Group 4: MTX drug loaded on GNPs in solution form, rats received MTX-GNPs IA at a dose of 0.1 mg/kg/day from day 14 till 28 one dose per week.
Group 5: MTX drug loaded on GNPs in gel form, 1 g MTX-GNP gel formula containing 0.1 mg active constituent applied transdermal from day 14 till 28 in a continuous manner (transdermal application of the gel every 12 h).
Group 6: CFA-MTX drug, rats received a combination of CFA and then were treated with MTX drug (0.1 mg/kg/day) on day 14 and day 28.
Group 7: CFA-MTX drug loaded on GNPs in solution form, rats received a combination of CFA, and then were treated with MTX-GNPs IA at a dose of 0.1 mg/kg/day from day 14 till 28, one dose per week.
Group 8: CFA-MTX drug loaded on GNPs in gel form, rats received a combination of CFA and then were treated with 1 g MTX-GNP gel formula applied transdermal from day 14 till 28 in a continuous manner (two doses per day every 12 h). CFA dose and MTX dose have been chosen based on a previously published study [48].

Serum and tissue samples were collected to assess the following measures after 24 h of MTX application. The serum anti-cyclic citrullinated protein antibody (ACCP) was measured and assessed by ELISA. The serum cytokines, interleukin 6 (IL-6), and interleukin 10 (IL-10) were evaluated by the ELISA method. The PCR method was used to quantify cartilage oligomeric matrix protein (COMP) in the tissue.

The receptor activator of the NFκB ligand (RANKL) was detected in tissue also using the Western Blot technique. In the meantime, the entire knee joints were dissected and examined histopathological.

### 3.5. Methods

#### 3.5.1. Induction of RA

The current study depends on a modified aggressive arthritis model with CFA (1 mg/mL Mycobacterium tuberculosis, heat killed in mineral oil) [49], where a dose of 0.4 mL of CFA was injected SC on the planter surface in each right hind limb of each rat every four days for twelve days [24].

#### 3.5.2. Blood Sampling

On the twenty-eighth day, 24 h following the last MTX dosage, whole blood in non-heparinized form was drawn from the animals using light thiopental anesthesia (85 mg per kilogram/i.p.) [50]. Then, this was centrifuged at a rate of 4000× *g* for 10 min. The clear serum was withdrawn and kept in a −80 °C freezer (Als Angelantoni Life Science, Italy) to assess serum ACCP, IL-6, and IL-10.

#### 3.5.3. Tissue Sampling

After cervical dislocation, the entire joints of the knee were dissected including fresh synovial tissues and put instantly in liquid nitrogen, then frozen, and stored at −80 °C before either Western Blot analysis for RANKL or PCR analysis for COMP were processed. From another side, the rats’ other knee joints were as soon as possible removed and kept in buffered paraformaldehyde at a concentration of 4%. At last we made decalcification with 10% EDTA, then all samples were embedded in paraffin, and stained in five μm sections with H&E stain (hematoxylin and eosin) for further examination under a microscope.

#### 3.5.4. Serum Levels of ACCP, IL-6, and IL-10

For the quantitative determination, we used the ELISA method according to the [51] method. The Bradford method depends on Genei, Bangalore, protein estimation kit. A plate reader was used to measure color absorbance in the OD range of 490 to 630 nm (Stat Fax 2200, Awareness Technologies, 2325 SW Martin Highway, Palm City, FL 34990, USA).

#### 3.5.5. Protein Extraction and Western Blot Analysis of RANKL

Based on the method described earlier by [52], whole knee joint proteins were isolated from knee joints, treated using RIBA lysis buffer, homogenization, and were kept constantly stirring for two hours at 4 °C. The lysate was then centrifuged at 16,000× *g* for 30 min at 4 °C to eliminate cell debris after being incubated in ice for 30 min. By using the Bradford assay, protein concentrations were calculated. Western Blot was performed using the approach outlined by [53]. Briefly, 25–50 μg protein from every group was isolated by 10% SDS-PAGE using a BioRad mini protein electrophoresis separator unit (USPTO) before being electrophoretically transported to a nitrocellulose membrane. We made protein blot blockage at room temperature for 1 h, with tris-buffered saline with tween 20 (TBST) buffer and 3% bovine serum albumin (BSA). After that, the membrane was treated with primary antibodies against RANKL, and following that, suitable horseradish peroxidase-conjugated secondary antibodies were used. Following the manufacturer’s recommendation, to the blot we applied the chemiluminescent substrate (ClarityTM Western ECL substrate—BIO-RAD, USA). Utilizing a CDD camera-based imager, the chemiluminescent signals were captured. We quantified the bands to B-actinbands using the ChemiDoc™ MP imaging System with Image Lab™ Software, version 5.1 (Bio-Rad, 170-8280) [50].

#### 3.5.6. RNA Extraction and (Q-PCR) (Real-Time Polymerase Chain Reaction)

In accordance with the manufacturer’s instructions [purity (A260/A280 ratio)], for each rat, total RNA from knee tissue homogenate was separated using Qiagen tissue extraction kit, then the absorbance was measured at 260 nm for quantification using spectrophotometry (dual wavelength Beckman, Spectrophotometer, JascoV530, Easton, MD, USA). For cDNA conversion, we used the total RNA (0.5–2 μg) utilizing high-capacity cDNA reverse transcription kit ferments. Quantitative determination of mRNA expression of target genes was carried out by RT-PCR analysis. The analysis was carried out according to the method of Livak and Schmittgen [54], utilizing an Applied Biosystem (StepOne™, Thermo Fisher Scientific, 168 Third Avenue, Waltham, MA, USA 02451) software version 3.1 under the following circumstances:

denaturation at 95 °C for 15 s; annealing at 60 °C for 1 min; extension at 72 °C for 1 min for 40 cycles; and final extension at 72 °C for 7 min. Finally, we made isolation for the PCR product on 1.5% agarose gel and stained it with ethidium bromide. Specific primers used for COMP are documented in Table 3. By the 2^−ΔΔCT^ method [55], we calculated the relative amount of mRNA. The primers’ relative amplification efficiencies were examined and found to be comparable.

#### 3.5.7. Histopathological Examination

H&E staining was used on transverse sections of knee joints which were kept in paraffin. A light microscope was used to examine the sections, and representative photographs were obtained.

#### 3.5.8. Statistical Analysis

Data were presented as means and standard deviation. ANOVA as a one-way analysis of variance and a multiple comparisons test, Tukey–Kramer, were then used using Graph Pad Prism software (Version6.04, La Jolla, CA, USA). *p* < 0.05 was considered significant.

## 4. Conclusions

As is significant, the results obtained from this current research have supported the efficacy of NPs in targeting varied drugs and bio-molecules. MTX was also successfully delivered via a GNPs nanotype. Our findings presented a remarkable improvement in the pharmacokinetics of MTX. However, further complementary investigations are needed to be applied in humans, by taking safety and efficacy issues into account. 

## Figures and Tables

**Table 3:** Primer sequence of all studied genes.

Gene Symbol	Primer Sequence from 5′–3′ F: Forward Primer, R: Reverse Primer	Gene Bank Accession Number
**COMP**	F: ACACAGGGTCAAGGAGATCAC	NM_012834.2
	R: AGACTACGCCAGGGAAGCA	
**β-actin**	F: TCCGTCGCCGGTCCACACCC	NM_031144.3
	R: TCACCAACTGGGACGATATG	

## Data Availability

Data are contained within the article.
